# Importance of electrode position for the distribution of tumor treating fields (TTFields) in a human brain. Identification of effective layouts through systematic analysis of array positions for multiple tumor locations

**DOI:** 10.1371/journal.pone.0201957

**Published:** 2018-08-22

**Authors:** Anders Rosendal Korshoej, Frederik Lundgaard Hansen, Nikola Mikic, Gorm von Oettingen, Jens Christian Hedemann Sørensen, Axel Thielscher

**Affiliations:** 1 Aarhus University Hospital, Department of Neurosurgery, Nørrebrogade, Aarhus C, Denmark; 2 Aarhus University, Department of Clinical Medicine, Palle Juul-Jensens Boulevard, Aarhus N, Denmark; 3 Danish Research Center for Magnetic Resonance, Copenhagen University Hospital Hvidovre, Kettegaards Allé, DK, Hvidovre, Denmark; 4 Department of Electrical Engineering, Technical University of Denmark, Ørsteds Plads, DK, Kgs. Lyngby, Denmark; University of Portsmouth, UNITED KINGDOM

## Abstract

Tumor treating fields (TTFields) is a new modality used for the treatment of glioblastoma. It is based on antineoplastic low-intensity electric fields induced by two pairs of electrode arrays placed on the patient’s scalp. The layout of the arrays greatly impacts the intensity (dose) of TTFields in the pathology. The present study systematically characterizes the impact of array position on the TTFields distribution calculated in a realistic human head model using finite element methods. We investigate systematic rotations of arrays around a central craniocaudal axis of the head and identify optimal layouts for a large range of (nineteen) different frontoparietal tumor positions. In addition, we present comprehensive graphical representations and animations to support the users’ understanding of TTFields. For most tumors, we identified two optimal array positions. These positions varied with the translation of the tumor in the anterior-posterior direction but not in the left-right direction. The two optimal directions were oriented approximately orthogonally and when combining two pairs of orthogonal arrays, equivalent to clinical TTFields therapy, we correspondingly found a single optimum position. In most cases, an oblique layout with the fields oriented at forty-five degrees to the sagittal plane was superior to the commonly used anterior-posterior and left-right combinations of arrays. The oblique configuration may be used as an effective and viable configuration for most frontoparietal tumors. Our results may be applied to assist clinical decision-making in various challenging situations associated with TTFields. This includes situations in which circumstances, such as therapy-induced skin rash, scar tissue or shunt therapy, etc., require layouts alternative to the prescribed. More accurate distributions should, however, be based on patient-specific models. Future work is needed to assess the robustness of the presented results towards variations in conductivity.

## Introduction

Glioblastoma multiforme (GBM) is a devastating brain cancer with an incidence of approximately 3-4/100.000 [1]. Standard therapy includes maximal safe resection of the tumor followed by radio-chemotherapy [2–8]. In addition, tumor treating fields (TTFields) are increasingly being used as a supplementary treatment modality for both recurrent and newly diagnosed GBM [9–12]. TTFields are low intensity (1–3 V/m) intermediate frequency (200 kHz) alternating electrical fields, which inhibit tumor growth. Clinical trials with TTFields have demonstrated promising results [13–19]. For recurrent GBM, the technology performs as well as best physicians’ choice chemotherapy but is associated with far less discomfort and adverse events [13]. For newly diagnosed GBM, TTFields increase the median overall survival by approximately five months when applied in addition to standard radio-chemotherapy [14,17,19–21]

TTFields are generated by two pairs of 3x3 electrode arrays placed on the scalp of the patient so that the fields induced by each pair are presumably orthogonal. Each pair of arrays is supplied by a portable and battery-powered current source carried by the patient. The sources are activated in sequence so when one source is active, the other is inactive and vice-versa. Each source has a repeated 50% square (on/off) duty-cycle of 2 s total duration, i.e. within one duty-cycle the source is active for 1 s, and then inactive for 1 s. During activation, each source induces a 200 kHz sinusoidal alternating field and the maximum peak-to-peak current delivered is 1.8A. The current level is controlled to maintain a skin temperature below 41°C. The therapeutic benefit depends on user compliance and the device “on-time” and the device should be active for at least 18 hours per day [22] (https://www.optune.com/content/pdfs/Optune_PIOM_8.5x11.pdf).

The mechanism of action of TTFields is believed to relate mainly to direct physical interference with the mitotic process, such as septin and tubulin assembly and also direct migration of polarized particles in dividing cells [23–25]. Previous *in vitro* studies have established a significant dependency between the intensity of the induced field and the degree of tumor growth reduction [23,24]. The lower threshold of inhibition was determined to be 100 V/m while field intensities higher than 225 V/m induced tumor regression. Given this significant dose-response relationship, it is clear that the distribution of TTFields inside the head, brain, and tumor plays an important role for the expected treatment benefit for each patient. Therefore, methods to quantify this distribution are highly warranted, and recent studies have described various approaches to achieve this goal using finite element (FE) methods [26–36]. In a recent study, MRI data from a GBM patient were used to produce an accurate head FE model allowing for precise representation of the individual anatomy and dielectric property distribution. The approach was used to investigate the potential enhancement of TTFields using craniectomy and skull remodeling surgery to create paths for current flow directly into the tumor. In another study, a head model was constructed from MRI data of a healthy individual. The model was modified to incorporate virtual pathologies by post-processing of the head mesh [36] and used to identify general factors affecting the distribution of TTFields, such as tumor position, the type of surrounding tissue, and the presence of central necrosis in the tumor.

Despite the increasing focus on understanding the biophysics of TTFields, several questions remain unanswered. In this study, we will utilize a virtual lesion FE approach similar to the one described above to systematically investigate the impact of electrode position on the TTFields distribution and the average dose of TTFields experienced by tumors spanning a large range of the hemispheric regions. We will investigate the impact of systematic rotation of the transducer array pairs around a central craniocaudal axis of the head to investigate optimal configurations and transducer array layouts for various tumor locations. As a novel and significant finding, we will show that an oblique array layout is superior to the standard left-right (LR) and anterior-posterior (AP) array layout for many tumor locations. Furthermore, our results will provide systematic and direct visual and quantitative information on the impact of electrode movement on the TTFields distribution. This information is highly important for clinicians and users who often have to adapt TTFields therapy and electrode layout to counter common challenges, such as therapy-induced skin rash beneath the electrodes. We hope to support users and clinicians to gain a better understanding of the TTFields therapy treatment and thereby aid treatment planning and optimize the clinical implementation of the technology.

## Methods

### Field calculations

We calculated the electric field distribution in a realistic model of a human head using a finite element (FE) approximation of the electric potential [26,27,33,35–38]. In general, the quantities defining a time-varying electromagnetic field are given by the complex Maxwell equations [39]. However, in biological tissues and at the low to intermediate frequency of TTFields (*f = 200kHz*), the electromagnetic wavelength is much larger than the size of the head and the electric permittivity ε is negligible compared to the real-valued electric conductivity *σ*, i.e. $\frac{\omega \varepsilon }{\sigma }\ll 1$, where *ω* = 2*πf* is the angular frequency [40]. This implies that the electromagnetic propagation effects and capacitive effects in the tissue are negligible, so the scalar electric potential φ may be well approximated by the static Laplace equation ∇∙(*σ*∇φ) = 0, with appropriate boundary conditions at the electrodes and skin [26,31,40,41]. Thus, the complex impedance is treated as resistive (i.e. reactance is negligible) and currents flowing within the volume conductor are, therefore, mainly free (Ohmic) currents. The validity of this approximation for TTFields has further been established by Wenger *et al*., 2015 [31], who showed that permittivity affects the intensity of the resulting field distribution in a realistic human head model by less than 2%. Similar observations were recently made by Lok *et al*., 2017 [33]. Therefore, we have adopted the simpler electrostatic approximation in this study. We would like to note that, while this approach is valid for the estimation of the macroscopic field distribution in the head volume conductor, capacitive effects of the cell membranes have to be taken into account when modelling the penetration of the external current flow into tumor cells on the microscopic level [30,42]. However, the latter is not the topic of this study. The FE approximation of Laplace’s equation was calculated using the SimNIBS software (www.simnibs.org) [43]. Computations were based on the Galerkin method [44] and the residuals for the conjugate gradient solver were required to be <1E−9. Dirichlet boundary conditions were used with the electric potential was set to (arbitrarily chosen) fixed values at each set of electrode arrays [45,46]. The electric (vector) field was calculated as the numerical gradient of the electric potential and the current density (vector field) was computed from the electric field using Ohm's law. The potential difference of the electric field values and the current densities were linearly rescaled to ensure a total peak-to-peak amplitude for each array pair of 1.8 A, calculated as the (numerical) surface integral of the normal current density components over all triangular surface elements on the active electrode discs. This corresponds to the current level used for clinical TTFields therapy by the Optune® device. The “dose” of TTFields was calculated as the intensity (L^2^ norm) of the field vectors. We assumed the modeled current to be provided by two separate and sequentially active sources each connected to a pair of 3x3 transducer arrays (see below). The left and posterior arrays were defined to be sources in the simulations, while the right and anterior arrays were the corresponding sinks, respectively. However, as TTFields employ alternating fields, this choice is arbitrary and does not influence the results.

### Head model generation and positioning of tumors and arrays

**Fig 1:**
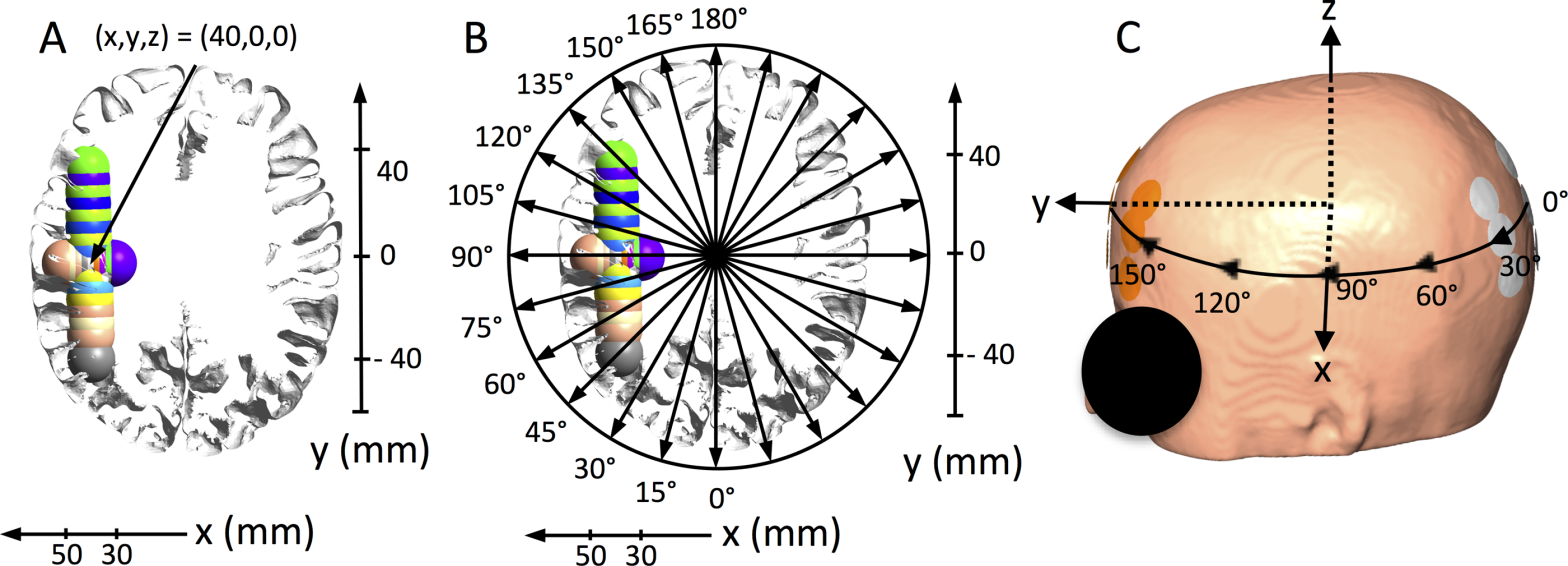
Visualization of coordinate system, tumor locations and electrode rotation. A. Axial section in the xy-plane of the GM and WM surface of the head model with all tumor locations superimposed (radiological orientation). X- and y-axes are shown to illustrate tumor center coordinates in millimeters. The tumor position x = 40 mm, y = 0 mm, and z = 0 mm is indicated by a solid arrow. All tumors were located in the electrode plane, i.e. z = 0 mm and had the following x- and y-coordinates: X-translations (mm): (30, 0, 0), (32.5, 0, 0), (35, 0, 0), (37.5, 0, 0), (42.5, 0, 0), (45, 0, 0), (47.5, 0, 0), (50, 0, 0). Y-translations (mm): (40, -40, 0), (40, -30, 0), (40, -25, 0), (40, -20, 0), (40, -10, 0), (40, 5, 0), (40, 10, 0), (40, 15, 0), (40, 20, 0), (40, 25, 0), (40, 30, 0). B. Same section as shown in panel A, but with illustrations of the tested electrode rotations in the xy-plane from 0 to 180 degrees at 15-degree intervals. C. Surface view of the head model with one electrode array pair in the AP position, i.e. 0 degrees. The x-, y-, and z- axes are shown along with a schematic illustration of the rotations path of the electrode arrays on the skin.

A realistic head model was constructed from MRI data from a healthy individual (almi5 dataset available at simnibs.org). The computational head mesh was initially segmented into skin, bone, cerebrospinal fluid (CSF), gray matter (GM) and white matter (WM). To ensure systematic positioning of tumors and electrode arrays, we defined a right-handed reference coordinate system in the model (Fig 1). We kindly refer the reader to Korshoej *et al*. (2017) for a full description of how this coordinate system was constructed [36]. In summary, a transversal plane was initially defined by conventional LR and AP positioning of the arrays. The left-right direction was defined as the x-axis, the AP direction as the y-axis, and the cranio-caudal direction normal to the xy-plane was defined as the z-axis.

After defining the coordinate system, nineteen spherical tumors were placed at systematically varying x, y and z coordinates to obtain a wide and clinically relevant range of cortical and subcortical tumor positions in the frontal, parietal, and occipital regions of the right hemisphere (Fig 1A). Tumor lesions were translated along the defined axes around a central position at the frontoparietal junction (corresponding to x = 40 mm, y = 0 mm, z = 0 mm, see Fig 1). The exact tumor coordinates are stated in the caption of Fig 1 and the intersecting surfaces of all tumors are shown in Fig 1A and 1B along with corresponding x- and y-coordinate axes (z = 0). Tumor lesions had external radii of 10 mm and inner core radii of 7 mm defining a core of central necrosis, as previously investigated by the Authors and by Miranda *et al*. [26,28,31].

Electrode arrays consisted of nine electrodes of 20 mm diameter arranged in a 3x3 array structure. The center-to-center distances between neighboring electrodes were 45 mm and 22 mm, respectively (Fig 1C). The transducer array configuration corresponded entirely to the Optune™ technology, which is used for clinical treatment. Transducer arrays were placed with their centers and longitudinal axes in the xy-plane. A pair of arrays was systematically rotated around the z-axis of the head model, i.e. in the xy-plane, from 0 to 180 degrees, thereby covering the entire circumference of the head (by symmetry). The rotation interval was 15 degrees, corresponding to approximately 2 cm translations, giving a total of twelve different positions in the range of 180 degrees (Fig 1C). Calculations were performed for electrode positions at all tumor locations.

Isotropic conductivity estimates corresponded to previous *in vivo* measurements at comparable frequencies [47–49]. Anisotropic conductivity tensor estimates were obtained for GM and WM using diffusion MRI and a linear direct mapping technique [50]. This mapping linearly rescales the diffusion tensors by a common factor to obtain the conductivity tensors. The scaling factor is chosen so that the geometric mean of the eigenvalues of the conductivity tensors fits as well as possible in a least-squares sense to isotropic reference values (0.276 S/m for GM, and 0.126 S/m for WM). For the remaining tissue types, the following isotropic conductivities were used: Tumor 0.24 S/m, necrosis 1.00 S/m, skin 0.25 S/m; bone 0.010 S/m; and CSF 1.654 S/m. These values are based on average values obtained from *in vitro* and *in vivo* experiments at comparable frequencies [51–54]. Additional detailed information about the methods used in this study can be found in Korshoej *et al*., 2016 [35], and 2017 [36].

## Results

### Effect of array rotation

We investigated the impact of array rotation separately for tumors whose positions were varied along the x- and y-axes, respectively. Two-dimensional color maps were created to visualize the effect of rotation for different tumor positions systematically along each of the two axes. As previously described, TTFields therapy in its current form (Optune™) is applied using two sequentially active array pairs oriented orthogonally to each other. This is done to distribute the effect across cells dividing in different directions because the effect is higher when the field is applied in the direction of cell division [23]. To assess clinically relevant layout configurations, we, therefore, combined the results of all sets of array pairs oriented orthogonally to each other by calculating the average field induced by the two orthogonal pairs in the tumor tissue. This is equivalent to calculating the average peak field in the tumor over one duty cycle of TTFields for the given configuration and allows for direct efficacy assessment of orthogonal array configurations at different rotations around the head. The results are presented as both color maps as well as visual field maps showing the strength of the induced field in representative sections of the head model.

### Tumors translated in the left-right direction (x-axis)

**Fig 2:**
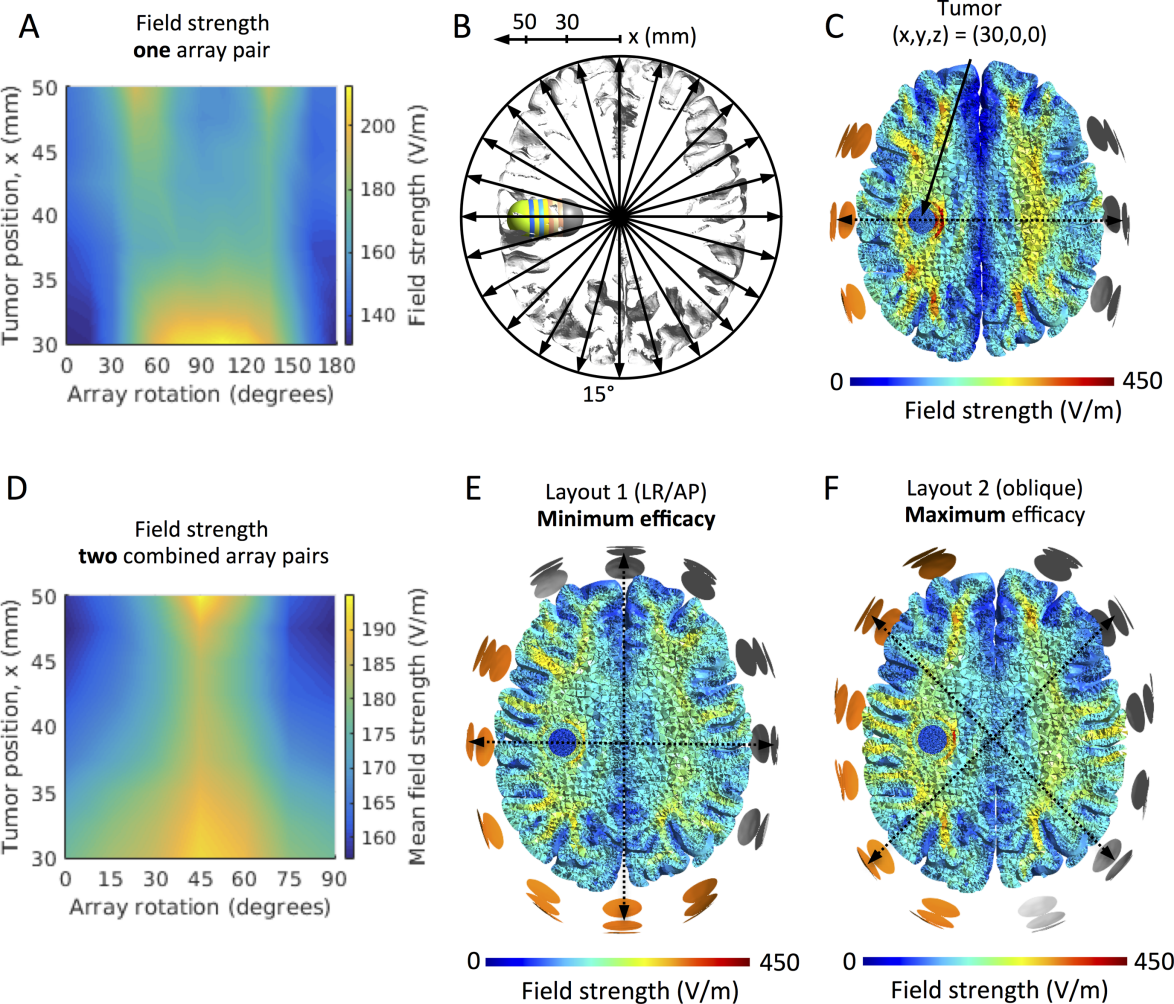
Effect of array rotation on field intensity for left-right tumor translations on the x-axis. A. Color map of the median field intensity (V/m) in the tumors at varying x-positions (30 mm to 50 mm, *ordinate*) and varying rotations (θ = 0 to 180 degrees, *abscissa*) of a single pair of electrode arrays. Y- and z-coordinates were kept constantly at zero for all tumors, i.e. all tumors were in the center-to-center plane of the rotated array pairs. The figure shows field maxima at θ = 45 and 135 degrees, respectively, for all tumors between x = 35 mm and 50 mm, while deeper seated tumors experienced high fields for all rotations between these values. B. Axial section of the GM and WM surfaces and the investigated tumors (x translations, i.e. x = 30 to 50 mm, y = 0 mm, and z = 0 mm). Array rotations and tumor locations are indicated by the corresponding arrows and axis, respectively. C. Axial section (radiological convention) of the WM, GM and tumor volume (x = 30 mm, position indicated by the solid arrow), showing an example of the topographical distribution of the field induced by TTFields (left-right array position, θ = 90 degrees). D. Color map comparable to panel A, but illustrating the mean field induced by two orthogonal array pairs. Tumor positions are indicated on the *ordinate* and the rotations of the posterior array on the abscissa (θ = 0 to 90 degrees). The figure shows a maximum mean field intensity at θ = 45 degrees equivalent to an oblique position of both pairs. The field distribution of this “optimal” layout is shown in panel F for the tumor position x = 30 mm, while the distribution of the least effective layout (θ = 0 degrees) for the same tumor is shown in panel E.

The median field intensity in the tumor varied considerably with tumor location and array position (Fig 2). For all tumor locations, the direct AP array position at θ = 0 and 180 degrees induced the lowest median field strength (E = 131 to 148 V/m). For tumors located in the cortical region, i.e. superficial to the sulcal fundi at x = 37.5 to 50 mm, the field intensity showed two maxima at the oblique orientations of θ = 45 degrees (E = 165–193 V/m) and θ = 135 degrees (E = 172–195 V/m), respectively. This phenomenon is illustrated in Fig 2A. The field was slightly lower (E = 155–170 V/m) for the LR array orientation, θ = 90 degrees. For tumors in the deeper subcortical regions (x = 30 to 35 mm), the field showed a smooth curve with a single maximum at θ = 90 degrees (E = 179–209 V/m). The field intensity at θ = 90 degrees increased for deeper tumor positions. Array positions at θ = 45 to θ = 135 degrees also induced high mean field intensities comparable to θ = 90 degrees. Fig 2C shows an example of the field distribution for the subcortical position x = 30 mm. The supplementary material S1–S3 Videos show additional animations of the changing field distributions for all array rotations at representative tumor locations encompassing both cortical and subcortical positions, i.e. x = 30 mm, x = 42.5 mm, and x = 50 mm, respectively.

The average field strength induced by two orthogonal pairs of arrays was maximal at θ = 45 degrees, *i*.*e*. *the combination of two oblique array pairs was superior to all other orthogonal combinations regardless of the tumor location along the x-axis* (Fig 2D). In addition, S4 Video shows an animation of the sequence of field distributions induced by the optimal combination of orthogonal array pairs as the tumor is gradually translated from medial to lateral. As it is evident, the optimal array position is the oblique layout (θ = 45 degrees and θ = 135 degrees) for all tumors along the x-axis. The average field for the oblique layout was between E = 183 V/m and E = 195 V/m depending on tumor position, and this corresponds to 9–23% enhancement compared to the least effective standard AP/LR layout at θ = 0 and 90 degrees. Fig 2E and 2F shows the topographical distribution of the average field for both the most effective array position θ = 45/135 degrees and the least effective θ = 0/90 degrees, respectively, at the tumor position (x = 30 mm). Animations of the average field distributions for *combined* orthogonal arrays pairs are shown in the supplementary material S4–S6 Videos for all rotations and the same representative tumor locations, x = 30 mm, x = 42.5 mm and x = 50 mm, as shown in S1–S3 Videos.

### Tumors translated in the anterior-posterior direction, y-axis

**Fig 3:**
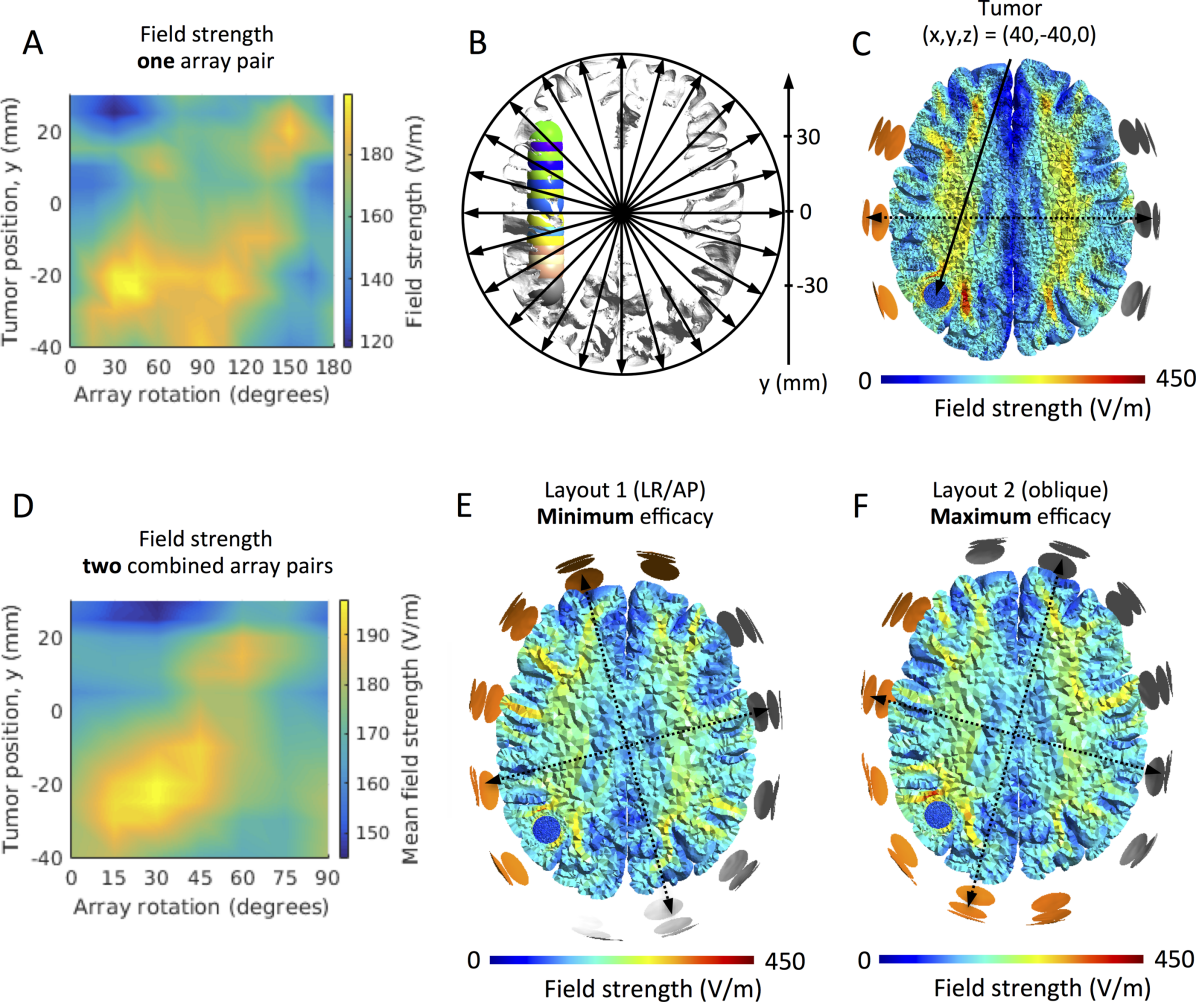
Effect of array rotation on field intensity for anterior-posterior tumor translations, y-axis. A. Color map of the median field intensity (V/m) in tumors at varying y-positions (-40 mm to 30 mm, *ordinate*) and varying rotations (θ = 0 to 180 degrees, *abscissa*) of a single pair of electrode arrays. X- and z-coordinates were kept constant at x = 40 mm and z = 0 mm, respectively, for all tumors, i.e. all tumors were in the center-to-center plane of the rotated array pairs. The figure shows field maxima at two separate rotations for the most tumors. The two maxima were separated by approximately 90 degrees. B. Axial section of the GM and WM surfaces and the investigated tumors (y translations, i.e. x = 40 mm, y = -40 to 30 mm, and z = 0 mm). Array rotations and tumor locations are indicated by the corresponding arrows and the axis, respectively. C. Axial section (radiological convention) of the WM, GM and tumor volume (y = -40 mm, position indicated with a solid arrow), showing an example of the topographical distribution of the field induced by TTFields (left-right array position, θ = 90 degrees). D. Color map comparable to panel A, but illustrating the mean field induced by two orthogonal array pairs. Tumor positions are indicated on the *ordinate* and the rotations of the posterior array on the abscissa (θ = 0 to 90 degrees). The figure shows a single maximum of the mean field intensity at varying rotations depending on the tumor of interest. The field distribution of the “optimal” layout is shown in panel F for the tumor position y = -40 mm, while the distribution of the least effective layout (θ = 0 degrees) for the same tumor is shown in panel E.

For most parietal and occipital tumors, i.e. y ≤ 0 mm, array positions between θ = 15 and θ = 135 degrees all tended to perform well and induced relatively high field values (Fig 3). For frontal tumors (i.e. y > 0 mm) there was a tendency towards higher field values for array positions at θ = 60 degrees and higher. However, looking more closely at Fig 3, we see that for most tumor positions in the anterior-posterior direction, there are two peaks in the plot of median field strength against different array rotations (Fig 3A), as it was also observed for cortical tumors translated in the left-right direction (see above). Correspondingly, the peak values also occurred at approximately orthogonal array positions. However, the array positions inducing peak field values varied with tumor location, as demonstrated in the oblique tendency of peak median field values in the color map. This observation is further illustrated in Fig 3D, which shows the average median field strength for all combinations of two orthogonal pairs of electrode arrays. The figure shows, that *for every tumor position*, *an optimum position for each pair of orthogonal arrays exists*. *Furthermore*, *the optimum position is gradually rotated as the tumor is moved in the anterior-posterior direction*. This concept is further animated in the supplementary material S8 Video, which shows the induced field distribution by the optimal array position for all tumors along the y-axis. As a general observation, for all the tumors the optimal layout was angled at approximately 45 degrees to the surface of the cortical region immediate overlying the tumor. Correspondingly, they were also oriented at approximately 45 degrees to the sulcal/gyral border in the vicinity of the tumor, as these were close to perpendicular to the brain surface. The field intensities induced by the optimum array layouts were generally 10% to 17% higher relative to the layout with the lowest efficacy. The oblique layout at θ = 45 degrees performed well for most tumor locations and better than the AP/LR layout (θ = 0 and 90 degrees) for all cases.

Fig 3C shows an example of the field distribution at the single array position (θ = 90 degrees) which induced maximum field intensity for a selected tumor at y = -40 mm. Correspondingly, Fig 3E and 3F show examples of the orthogonal combination of electrode layouts with the highest (E = 183 V/m) and lowest mean field intensity (E = 167 V/m) for the same tumor location, respectively. The supplementary material S9–S12 Videos, shows animations of the field distributions induced by single rotating array pairs for tumor locations spanning a large (7 cm) anterior-posterior range, i.e. y = -40 mm, y = -40 mm, y = -20 mm, y = 20 mm, and y = 30 mm. The corresponding distributions of *combined* orthogonal arrays pairs (mean field intensity) are shown in S13–S16 Videos for the same tumor positions, respectively.

## Discussion

In this study, we used realistic head models and finite element calculations to compute the distribution of TTFields for a large number of transducer array layouts and tumor positions. Specifically, we have investigated the impact of systematic rotation of a pair of transducer arrays around a central craniocaudal axis of the head on the dose of TTFields in nineteen systematically varied tumor positions. We have identified optimum array positions for each tumor position and presented elaborate animations and graphical representations to provide users with a more intuitive understanding of the effect and distribution of TTFields and how this depends on the tumor and electrode array position.

As general observations, we found that varying the array layout produced markedly different field distributions and that the array position had a great impact (up to 23%) on the median field intensity observed in the tumor region for all studied tumor positions. In particular, when varying the tumor from left to right (Fig 2A), there was a bimodal dependency between the field intensity in the tumor and the array position, meaning that the field dose peaked at two separate (optimal) array positions. This result was observed for most superficial tumor positions in the cortical regions. In addition, the two optimal array positions were close to orthogonal for most tumors, i.e. positioned at ninety-degree intervals to each other. In the deeper subcortical regions, we observed a single optimum array position at which the field was maximal (θ = 90 degrees), however, high field intensities were observed for a wide range (45 to 135 degrees) of electrode positions, and the optimum combination of two orthogonal field pairs had a single peak at θ = 45 degrees as observed for the superficial tumors on the x-axis.

When varying the tumor position in the anterior-posterior direction, we observed a significant dependency between the optimum array configuration and the tumor position. Specifically, the optimum position of the orthogonal pair of arrays was gradually shifted clock-wise as the tumor was moved from posterior to anterior positions (viewed in radiological convention). However, array positions between θ = 15 and 135 degrees were all relatively effective for most parietooccipital tumors, while rotations of θ = 135 degrees or higher were generally more effective for frontal tumor locations.

**Fig 4:**
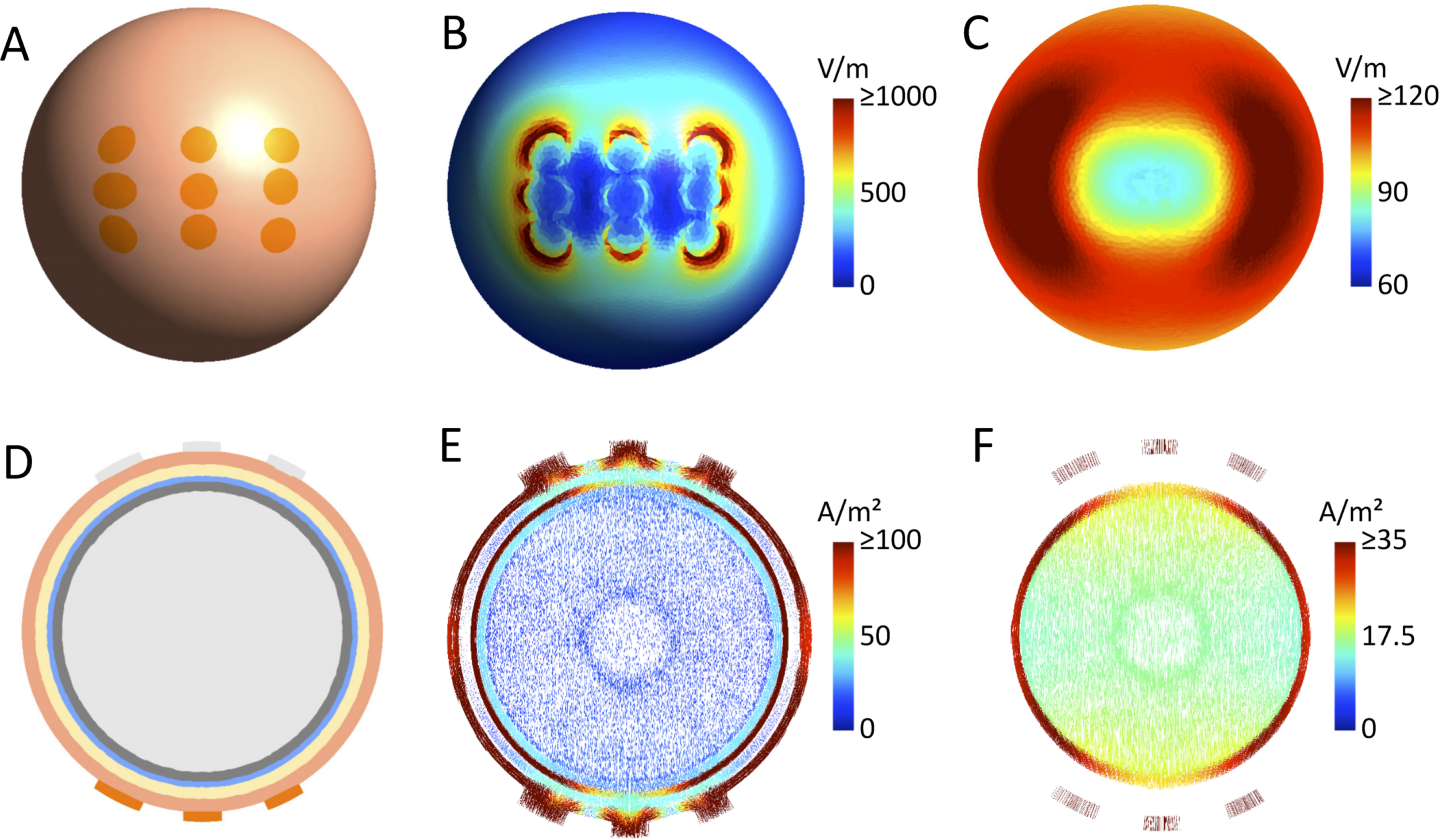
”Edge” effects for 3x3 electrode arrays. **A.** One of the two electrode arrays seen from above. **B.** Electric field distribution on the skin surface. The higher field strengths at the outer edges of the electrode array are clearly visible. **C.** Electric field distribution on the GM surface. The distribution is smoother than on the skin surface, but the lower field strengths underneath the array center are still clearly observed. **D.** Cut through the spherical head model. It consists of a central WM sphere (r = 7.5 cm), surrounded by GM (outer r = 8.0 cm), CSF (outer r = 8.3 cm), skull (outer r = 8.9 cm) and skin (outer r = 9.5 cm). The same tissue conductivities as for the head model were used, see Methods. GM and WM were modelled as isotropic with σ_GM_ = 0.276 S/m and σ_WM_ = 0.126 S/m. **E.** Current flow in the plane shown in D. The lower current densities in the skin and CSF layers underneath the central electrode are clearly observable (the different arrow densities in the sphere center are an artefact of the meshing process and do not influence the results). **F.** Current flow in GM and WM in the same plane. The lower current densities in the GM layer underneath the central electrode are clearly visible.

Due to the size of the electrode arrays, superficial tumors in our simulations were close to one of the outer electrode columns of an array for the oblique array orientations. It is interesting to note that the fields in the more superficial brain regions tend to be stronger underneath the outer electrodes than the central electrode of an array (see, e.g. S3 Video). This resembles the well-known edge effect for large pad electrodes used in transcranial electric brain stimulation [55], due to which the field is higher underneath the edges compared to the center of these electrode pads. Here, given that all electrodes of a TTFields electrode array are connected to the same channel and thus have the same electric potential, the same effect occurs and causes an unequal distribution of the current strength across the electrodes, with the outer electrodes tending to induce stronger currents into the underlying skin area (Fig 4 shows this exemplarily for a spherical head model). This effect is influenced by the local tissue conductivities underneath the electrodes (e.g., thin vs. thick skull) and thus varies across array orientations. However, the simulation results indicate that it is observed for the majority of orientations. As a consequence, when employing orthogonal pairs of arrays at an oblique orientation, superficial tumors will be close to an outer electrode column of one of the arrays for each of the two pairs, resulting in high median field strength. This explains the superiority of the 45° layout compared to the standard LR and AP electrode montages for the superficial tumor positions when varying the position from left to right. This effect is further increased by the generally low field intensity caused by the AP electrode array pair (0 and 180 degrees in Fig 2A; please refer to Korshoej *et al*. (2017) for a discussion of the reasons underlying the differences between LR and AP). The enhanced field intensities underneath the outer electrode columns also explain the gradual shift of the optimal array orientation when varying the tumor position from posterior to anterior. This is exemplified by the superficial tumor location x = 50 mm (y = 0 mm and z = 0 mm), which experienced the strongest fields at 45 degrees and 135 degrees, when the tumor was closest to the anterior and posterior electrode columns, respectively, S3 Video. The reduction of field intensity at angles between the two maxima (minimum at the left/right array position, Fig 2A) may also be explained in part by the edge effect because lower currents are induced underneath the central electrode. A likely contributing factor, however, is the fact that increasing amounts of current are shunted through the sulci and thus pass the tumor when the field is oriented increasingly in parallel with the sulci, i.e. close to the LR position.

The LR and AP configuration is a commonly employed layout for a wide range of tumors *In this study*, *we find that the vast majority of the investigated cases would benefit more from the oblique configuration (45 degrees to the sagittal plane) than from the LR/AP array configuration*, *although even further optimization may be performed in accordance with the above results*. *Specifically*, *the field was maximized when the neighboring arrays were orthogonal and placed such that their outermost columns of electrodes were close to the tumor*. We found that each tumor position was associated with a single optimum position of two orthogonal array pairs, at which the induced field was highest. From a treatment efficacy point of view, the ninety-degree relationship between optimal array positions (bimodality, Figs 2A and 3A) is highly convenient. This is because clinical TTFields therapy is performed with two orthogonal pairs of arrays in order to target cells with a different (random) direction of mitosis and distribute the tumor-inhibiting effect across larger regions of the pathology [23].

In general, our observations support the notion that the dose of TTFields, and therefore expectedly also the therapeutic efficacy, depends significantly on the positioning of transducer arrays on the scalp. Under normal circumstances, a personalized layout is produced for each patient prior to treatment initiation, using the software NovoTAL™ (Novocure, Ltd.). Very often, however, clinicians are faced with the challenge of having to deviate from the optimum layout due to various circumstances, such as scar tissue, use of metallic bone-fixation implants following surgery, or shunt therapy, etc. In addition, patients are recommended to move the arrays by approximately 2 cm when they are changed every 3–4 days. This is done to minimize the risk of skin rash beneath the electrodes, which is a commonly observed side effect of TTFields therapy. Our results may be used to support clinicians and users in deciding optimal array layouts for patients undergoing therapy, when facing challenges as described above. Of the tumors investigated, many would be treated with the LR and AP array layouts. This layout is produced from FE calculations of the field distribution, based on field head models adapted to the individual head and tumor morphometrics comparable to the approach used here. Our results suggest that some patients may in fact benefit more from the oblique array layout or layouts adapted in accordance with the above. In any case, the results presented provide viable alternative layouts for a wide range of tumors.

### Limitations and future perspectives

It is important to highlight that the investigated model reflects a limited, but comprehensive variety of scenarios specific for the individual case. Although a large number of tumor and array positions were investigated, personalized modeling experiments should ideally be conducted to account for the complex individual variations in anatomy and (as far as possible) tissue conductivities, including the shape and size of the head and tumor, observed in real tumor patients. Personalized approaches may account more accurately for the expected treatment efficacy and support more precise, safe and efficacious treatment planning. In addition, personalized approaches based on new and representative MRI data would enable a better and timelier characterization of the TTFields distribution at the moment of interest. Thereby the model would better account for changes, e.g. in tumor size or tissue composition, which may occur during the course of TTFields treatment as a result of disease development or therapy. Recently, the authors published an example of an individualized approach to TTFields modeling.

It should also be emphasized that the ohmic conductivity estimates used in the model markedly influence the results of the field calculations (see *Methods*). Recent studies by Wenger *et al*., 2015 [31], and Lok *et al*., 2017 [33], assessed the sensitivity of TTFields FEM models, such as the one employed in this study, towards dielectric property variations. They conclude that the conductivity variations significantly influence the distribution of TTFields, while permittivity variations only play an insignificant role. In this study, we thus neglected the influence of permittivity and have employed common conductivity values taken from the literature. However, it should be emphasized that the biological tissue conductivities have not been firmly established at 200 kHz, so that the resulting uncertainty might impact the generalizability of our results. However, since the field changes observed with changing tumor and electrode position were primarily associated with relative differences in conductivity between brain tissue, tumor tissue, and CSF [36], the observations reported will likely hold for a range of conductivity variations, as long as the differences in conductivity are in the same direction, i.e. the conductivity of CSF is higher than the conductivity of tumor, and the conductivity of tumor is higher than the conductivity of brain tissue. Although the absolute field values will change with varying conductivities, we expect that our main findings and conclusions will hold for a wide range of scenarios.

In addition to field intensity, future models should ideally also incorporate information about exposure time and the amount of non-orthogonality (i.e., directional correlation) of the electric fields that are induced by the two electrode array pairs in the tumor. The latter aspect relates to the finding that the effects of TTFields on a dividing cancer cell are enhanced when the field is aligned with the direction of cell division. Given that this direction varies randomly across tumor cells, treatment efficacy seems to be improved when two or more oblique fields rather than a single field direction are used [23]. Also, future models should incorporate better segmentations of the pathology to obtain representative field estimates in the true regions of interest. Finally, it is important to highlight that future consideration should also be given to validating the simulation results by means of direct TTFields measurements in vivo.

## Conclusion

We present novel findings, describing the impact of systematic positional variation of TTFields electrode arrays on a human head for a large number of tumor locations in the brain. For most superficial tumor positions, we found that there were two optimal array positions, at which the median field intensity was highest. These varied systematically (i.e. clockwise rotation), as the tumor position was translated from posterior to anterior, while it was unaffected by left-right tumor translations. In addition, the two optimal layouts were oriented approximately orthogonally to each other. Correspondingly, we found that there was a single optimal layout of combined orthogonal array pairs, at which the median TTFields dose was highest. The optimum position also varied systematically in accordance with the above and in general, the optimum arrays were oriented at 45 degrees to the surface of the brain immediately overlying the tumor. We also found that one particular layout was more effective for most tumor locations compared to the standard LR and AP combination of array pairs. This layout was composed of two orthogonal and oblique array positions both oriented at 45 degrees to the mid-sagittal plane. The presented results may generally guide layout configuration in clinical cases where deviations from the suggested NovoTAL layout are required, e.g. due to therapy-induced skin rash, etc. Furthermore, the oblique configuration may potentially be used as an effective, alternative layout for most tumors although further optimization may be expected by direct comparison with tumor representative locations. In addition, our results present extensive animations and graphical representations, to illustrate the impact of tumor and electrode positions on the distribution of TTFields. Our results will hopefully improve users’ understanding of TTFields and support clinical decisions on TTFields therapy.

The modeling approach used in this study is widely adopted. However, it must be noted that variations in tissue conductivities and head morphology are likely to affect the results. Further studies are required to estimate the robustness and sensitivity of the suggested conclusions towards these variations. Also, accurate field estimation should ideally be computed from patient-specific head models, which accurately represent the anatomy and tissue conductivity of the given individual.

## Supporting information

S1 Video Field distribution for rotation of a single array pair at the tumor position x = 30 mm. (MP4)

S2 Video Field distribution for rotation of a single array pair at the tumor position x = 42.5 mm. (MP4)

S3 Video Field distribution for rotation of a single array pair at the tumor position x = 50 mm. (MP4)

S4 Video Field distribution of optimal array position for all tumors translated in the left-right direction. (MP4)

S5 Video Field distribution for orthogonal array pairs at various rotations and tumor position x = 30 mm. (MP4)

S6 Video Field distribution for orthogonal array pairs at various rotations and tumor position x = 42.5 mm. (MP4)

S7 Video Field distribution for orthogonal array pairs at various rotations and tumor position x = 50 mm. (MP4)

S8 Video Field distribution of optimal array position for all tumors in the anterior-posterior direction. (MP4)

S9 Video Field distribution for rotation of a single array pair at the tumor position y = - 40 mm. (MP4)

S10 Video Field distribution for rotation of a single array pair at the tumor position y = -20 mm. (MP4)

S11 Video Field distribution for rotation of a single array pair at the tumor position y = 20 mm. (MP4)

S12 Video Field distribution for rotation of a single array pair at the tumor position y = 30 mm. (MP4)

S13 Video Field distribution for orthogonal array pairs at various rotations and tumor position y = -40 mm. (MP4)

S14 Video Field distribution for orthogonal array pairs at various rotations and tumor position y = -20 mm. (MP4)

S15 Video Field distribution for orthogonal array pairs at various rotations and tumor position y = 20 mm. (MP4)

S16 Video Field distribution for orthogonal array pairs at various rotations and tumor position y = 30 mm. (MP4)
